# Effects of Osteoglycin (OGN) on treating senile osteoporosis by regulating MSCs

**DOI:** 10.1186/s12891-017-1779-7

**Published:** 2017-10-26

**Authors:** Xia Chen, Junsong Chen, Dongliang Xu, Shuangxia Zhao, Huaidong Song, Yongde Peng

**Affiliations:** 1 Department of Endocrinology and Metabolism, Shanghai General Hospital of Nanjing Medical University, 100 Haining Road, Shanghai, 200080 China; 2 Key Laboratory of Systems Biomedicine(Ministry of Education), Shanghai Center for Systems Biomedicine, Shanghai Jiao Tong University, 800 Dongchuan Road, Shanghai, 200240 China; 3 Department of Urology, Shanghai General Hospital, Shanghai Jiao Tong University School of Medicine, 100 Haining Road, Shanghai, Shanghai 200080 China; 4 Shanghai Ninth People’s Hospital, Shanghai Jiaotong University School of Medicine, No. 639 zhizaoju Road, Shanghai, China

**Keywords:** Osteoglycin, Peroxisome proliferators-activated receptor-γ 2, Senile osteoporosis, Adipogenesis, Osteoblastogenesis, Mesenchymal stromal cells

## Abstract

**Background:**

Significant amount of bone mass is lost during the process of aging due to an imbalance between osteoblast-mediated bone formation and osteoclast-mediated bone resorption in bone marrow microenvironment, which leads to net bone loss in the aging population, resulting in the pathogenesis of osteoporosis.

**Methods:**

Firstly, differences in proliferative capacity of adipocyte or adipogenic differentiation in mouse mesenchymal stem cells (MMSCs) and senile mouse model-derived bone marrow mesenchymal stem cells (SMMSCs), as well as mRNA expression of OGN and PPARγ2 were observed. Secondly, osteogenic abilities of MMSCs and SMMSCs treated with rosiglitazone (a PPARγ2 agonist) to induce osteogenic changes were observed, and negative correlation of PPARγ2 with OGN was evaluated. Thirdly, the role of SMMSCs in promoting osteogenesis was examined through enhancing expression of OGN; besides, the related mechanism was investigated by means of expression of related adipocyte and osteoblast specific genes.

**Results:**

Forced OGN expression by OGN-infected lentivirus could increase expression of Wnt5b, RUNX2, OCN, ALP and Colla1, as well as bone formation, while decreases expression of adipogenesis marker PPARγ2. It resulted in expression inhibition of adipocyte genes such as adipocytic differentiation related genes adipocyte binding protein 2 (aP2) and osteoclast differentiation factor Rankl in bone marrow, giving rise to increased bone mass.

**Conclusion:**

OGN may plays a significant role in osteoporosis, which may also provide a potential target for therapeutic intervention of senile osteoporosis characterized by altered differentiation of BMSCs into osteoblasts and adipocytes.

## Background

Senile osteoporosis, which is defined by low bone mass and micro-architectural deterioration of bone tissue, leads to increased bone fragility and susceptibility to fracture [1, 2], thus becoming a severe societal problem threatening human health. Significant amount of bone is lost during the process of aging, which can be attributed to an imbalance between osteoblast-mediated bone formation and osteoclast-mediated bone resorption in bone marrow microenvironment. It leads to net bone loss in the aging population, resulting in the pathogenesis of osteoporosis [3, 4]. The reasons accounting for such imbalance inform us of the knowledge of senile osteoporosis.

Multiple endogenous and exogenous factors are proved to be involved in regulating bone remodeling, suggesting that both genetic and environmental factors are linked to bone mass and susceptibility to osteoporosis [5, 6]. However, the key regulating factor and the underlying mechanism of senile osteoporosis remain to be clearly defined. It is shown in a number of recent studies that deficiency in number and function of osteoblast, together with increase in marrow adipogenesis, may account for the key etiological factor of osteoporosis [7, 8]. Bone marrow-derived multipotent mesenchymal stromal cells (BMSCs) in the marrow pool are the major source of adipocytes and osteoblasts, which contribute to bone remodeling in adults. MSCs have the plasticity to differentiate into either osteoblasts or adipocytes; as a result, unbalanced differentiation of MSCs into marrow adipocytes and osteoblasts can result in bone loss by the excessive accumulation of marrow adipocytes [9]. Consequently, understanding factors regulating the osteogenic differentiation of BMSCs, as well as controlling the differentiation of MSCs to stimulate osteoblastogenesis while inhibit adipogenesis marks an effective way to treat senile osteoporosis. Studies on peroxisome proliferators-activated receptor-γ (PPARγ), a master regulator of adipocyte differentiation [10], support this hypothesis at least partly due to suppression of osteogenic differentiation of MSCs. PPARγ1 is widely expressed, particularly in adipose, liver, heart, and spleen; whereas expression of PPARγ2 is largely restricted to adipocytes [11].

Osteoglycin (OGN) may be one of the muscle-derived humoral bone anabolic factors [12], the level of which together with effects of conditioned medium on OGN-modulated myoblasts is positively correlated with phenotype and mineralization of osteoblasts [13]. Therefore, OGN can be served as a common factor to regulate the directional differentiation of MSCs, and a novel target of therapy for senile osteoporosis. This research aimed to carry out an intensive study on the regulatory effect of OGN on osteoblastogenesis and adipogenesis of MSCs, influence of inhibiting the adipogensis of MSCs by OGN on the function of osteoblasts and osteoclasts, as well as the molecular mechanisms by which OGN controlled differentiation of MSCs.

## Methods

### Materials

#### Cell culture

Mouse mesenchymal stem cells (MMSCs) were derived from bone marrow of femur and humerus in C57BL/6 mice (MUBMX-01001, Cyagen Biosciences Inc., CA. US), which had the potential to develop into mature cells that produced fat, cartilage, bone, tendon, and muscle. MMSCs were cultured in OriCell Mouse Mesenchymal Stem Cell Growth Medium (Cat. No. MUXMX-90011, Cyagen Biosciences Inc. CA. US) and maintained at 37 °C in a humidified atmosphere with 5% CO_2_. MMSCs have been passaged no more than 3 times. PPARγ2 selective agonist Rosiglitazone for cell culture experiments was purchased from Abcam (ab120762, USA), dissolved in DMSO to prepare the stocking solution with concentration of 40 mM, and stored at −20 °C. Part of cells received no treatment (Naïve) while the remaining cells were treated with vector (DMSO, with the final concentration of 0.1%). Relevant protein or mRNA expression and activation, as well as accumulative expression of fat droplets were analyzed 14 days after cell culture with drug treatment.

Primary bone marrow cell cultures were prepared from senescence accelerated mouse prone/6 (SAM-P6, female) of 4–5 months of age were acquired from Shanghai Tanhui Bio Co. Ltd. China. All animal studies were reviewed and approved by the Institutional Animal Care and Use Committee of Nanjing Medical University, and SAM-P6 mice were housed in a temperature-controlled (kept at 24 ± 1 °C) room with a 06.00–18.00 h light cycle. Full details of the study approval can be found under the approval ID, 20,140,871. After mice were anesthetized using isoflurane and killed by cervical dislocation, one femur and two tibiae were aseptically dissected and soft tissues were removed, and then the bone were put in phosphate buffered saline (PBS). Marrow was flushed from the femur and tibia, and was strained using a 70 μm cell strainer. Subsequently, it was suspended in MSC medium consisting of α-minimal essential medium (MEM, which contained 10% fetal bovine serum (FBV) and 100 U/ml penicillin), 100 μg/ml streptomyocin, and 0.25 μg/ml Amphotericin-B. Medium was replaced three times per week and the cells have been passaged no more than 3 times.

#### Osteogenic differentiation

MMSCs and primary bone marrow cells from senescence accelerated mouse model (SMMSCs) were cultured in osteogenic differentiation medium (Cyagen, Santa Clara, CA, USA) for 2 weeks, so as to induce osteogenic differentiation, with medium being replaced every 3 days. Osteogenic medium was comprised of 10% FBS (Hyclone), 100 U/ml penicillin, 100 mg/ml streptomycin, 0.1 mM dexamethasone, 0.2 mM ascorbate and 10 mM β-glycerophosphate (Sigma-Aldrich)).

#### Adipogenic differentiation

MMSCs and SMMSCs were cultivated in adipogenic differentiation medium consisting of DMEM-low glucose (Gibco) that was supplemented with 10% FBS (Hyclone), 1% penicillin-streptomycin, 1 mM dexamethasone, 0.5 mM methyisobutylxanthine, 100 μM indomethacin, and 10 mg/ml insulin (all Sigma-Aldrich) for the first 3 days, and the medium was replaced every 3 days. Six days later, cells were cultured in maintenance medium consisting of 10% FBS, 1% penicillin-streptomycin and 5 mg/ml insulin. Cultures were alternated weekly between differentiation medium and maintenance medium for two more weeks.

### Alizarin red staining, ALP staining and quantitative assay of osteogenesis

Alizarin red staining and ALP staining on MMSCs and SMMSCs were performed according to instructions from manufacturer (2% aqueous solution, Sigma-Aldrich, St. Louis, MO, USA), so as to detect mineral deposition. Briefly, cells were rinsed and fixed in 4% formaldehyde for 30 min at 4 °C. Subsequently, cells were washed with distilled water, exposed to Alizarin red or ALP for 20 min at room temperature, and washed again with distilled water for 5 min for 4 times. Quantitative analysis was conducted after Alizarin red staining by means of optical density value at 450 nm, and discs without cells were also stained as controls. ALP activity assay was performed in accordance with instructions from manufacturer. Briefly, culture solution on 14th day of subculture was collected from the 6-well plates, and quantitative detection of ALP levels was thereby carried out using poly-biochemical analyzer. Each experiment was performed in triplicate. Eventually, cells were observed and photographed under phase-contrast microscopy.

### MTT and colony formation assay

Cells at the concentration of 1 × 10^3^/well were seeded into the 96-well culture plates for MTT assay. Cell viability was assessed using 3-(4,5-dimethylthiazol-2-yl)-2, 5-diphenyl tetrazolium bromide (MTT, Sigma) dye in accordance with the protocol and the incubation time recommended by manufacturers. Amount of MTT formazan product was analyzed using spectrophotometry at a wavelength of 490 nm (Bio-Rad). Each individual experiment was repeated for at least 3 times.

Colony formation assays were performed in the 6-well culture plates at the cell seeding density of 1000. Cells were washed with PBS and fixed with methanol at room temperature for 20 min after 14 days. Colonies were stained with 0.1% crystal violet (Sigma) and counted. The cultures were replaced twice weekly, and colonies of more than 30 cells were scored. All experiments were conducted in triplicate.

### Lipid accumulation and quantitative assay of adipogenesis

Oil Red O staining (Sigma, St. Louis, MO, USA) was performed to detect lipid accumulation in adipocytes. Cultures were fixed in 10% phosphate buffered formalin for 10 min. Cells were rinsed with PBS once for 1 min, followed by 60% isopropanol for 5 min, so as to facilitate the staining of neutral lipids. Subsequently, cells were stained with an aqueous filtered solution of Oil Red O at 37 °C for 30 min in darkness. Later, cells were destained with 60% isopropanol for 10 min, and rinsed with PBS for 3 min for 3 times altogether. Cultures were magnified through light microscopy (Olympus) and photographed; in addition, lipid accumulation was quantified by Nile Red staining.

### Quantitative real-time RT-PCR (qRT-PCR)

Total RNA was extracted and genomic DNA was removed with TRIzol (Invitrogen, Carlsbad, CA, USA). cDNA was synthesized by total RNA using Superscript II Reverse Transcriptase (Invitrogen) according to the recommendations from manufacturers. Quantitative RT-PCR (qRT-PCR) was performed by adopting FastStart Universal SYBR Green Master with LightCycler 2.0 real-time PCR system (Roche Germany). Validated primers were purchased from Biosystems (as was shown in Table 1). Relative transcript levels were analyzed in a 20 μL reaction system on 96-well plates using a BIORAD CFX96 real-time PCR system. The reaction conditions were shown as follows: Hot-Start activation at 95 °C for 2 min, and 40 cycles of denaturation (at 95 °C for 16 s) and annealing/extension (at 54 °C for 65 s). β-actin was used as an internal reference. Expression levels of PCR products of interest relative to those in the naïve cultures (control groups) were calculated on the basis of relative quantitative method (2^ΔΔCT^), and the data were expressed as “Relative expression Vs. day 0”. All reactions were conducted in duplicate, and all experiments were repeated for at least 3 times.

**Table 1:** Primers of genes

Gene (mouse)		DNA sequence (forward/reverse, 5′-3′)
PPARγ2	Forward	ATGGGTGAAACTCTGGGAGA
	Reverse	GAGCTGATTCCGAAGTTGGT
OGN	Forward	GGCGCTACCTGTATCAATGG
	Reverse	TCAGCCAACTCGTCACAGTC
ALP	Forward	GTGCAGTCTGTGTCTTGCCTG
	Reverse	CCTTGCCTGTATCTGGAATCCT
RUNX2	Forward	CACCGAGACCAACCGAGTCA
	Reverse	TGCTCGGATCCCAAAAGAAG
Col1a1	Forward	CAACCTGGACGCCATCAAG
	Reverse	ATCGGTCATGCTCTCTCCAAA
OCN	Forward	AGCTTAACCCTGCTTGTGACG
	Reverse	GGAGGATCAAGTCCCGGAGA
Rankl	Forward	CACCATCAGCTGAAGATAGT
	Reverse	CCAAGATCTCTAACATGACG
aP2	Forward	CACCGCAGACGACAGGAAG
	Reverse	GCACCTGCACCAGGGC
Wnt5b	Forward	CTGCTGTTTTGAGGGGATTC
	Reverse	CGCACTGAGCAATTAAGCAG
β-actin	Forward	GTTGTCGACGACGAGCG
	Reverse	GCACAGAGCCTCGCCTT

### Lentivirus production

Human cDNA of OGN was subcloned into the pLJM1-EGFP lentiviral vector (Plasmid #1931, Addgene diagnostic digest). Viral vector, the expression of which was highly cell specific (target gene), was transfected into HEK293T cells in accordance with the instructions from manufacturers (Addgene). Viral supernatants were collected after 48 h, centrifuged at 1500×*g* for 5 min, filtered with a 0.45 μm filter, aliquoted, and stored at −80 °C. Viral titer was determined by serial dilution and infection of SMMSCs. SMMSCs, which were isolated from SAM-P6 mouse, were infected with empty control vector pLJM1-EGFP for 8 h, respectively. Cells were screened out by puromycin for 8 days after 2 days of infection, the resistant clones of which were pooled and confirmed as OGN-positive SMMSCs by Western blotting and EmGFP for easy determination of lentiviral titer by flow cytometry (data not shown).

### Western blot analysis

Cell lysates containing 30 μg of protein were separated by SDS-PAGE and transferred to activate PVDF membranes (Millipore Corp, Bedford, MA) that were blocked in defatted milk (5% in Tris-buffered saline with TWEEN-20 buffer) for 1 h and incubated with antibodies. These antibodies were shown as below: anti-OGN (Osteoglycin Antibody (K-14), sc-47,277, Santa Cruz Biotechnology, Inc., CA); anti-PPAR gamma EP4394(N) (ab191407, abcam, US); anti-RUNX2 (EPR3099, ab92336, abcam, US); anti-aP2/Fabp4 (2120S, Cell Signaling Technology, US); anti-Rankl (ab45039, abcam, US); anti-Wnt5b (ab93134, abcam, US); anti-Osteocalcin (ab13420, abcam, US); anti-Alkaline Phosphatas (ab108337, abcam, US) and anti-β-actin (ab8227, abcam, US), with the last one being served as a loading control. In the following steps, blots were incubated in horseradish peroxidase-conjugated secondary antibodies (Santa Cruz), and developed using a chemiluminescence detection system (Millipore) after washing. Protein bands were analyzed on the basis of an image analysis system (Bio-Rad).

### Statistical methods

Statistical analyses in this research were performed adopting Prism 5 (GraphPad SoftwareInc., La Jolla, CA) and Student’s t-test was utilized for analyzing difference between the experimental groups and control group. Besides, Bonferroni correction was also used in which multiple comparisons were made. Differences were considered as statistically significant when *P* values were less than 0.05. Data were expressed as means ± standard deviation.

## Results

### Compared with MMSCs in vitro, proliferation and osteogenic differentiation of SMMSCs were impaired, but adipogenic differentiation was enhanced

**Fig. 1:**
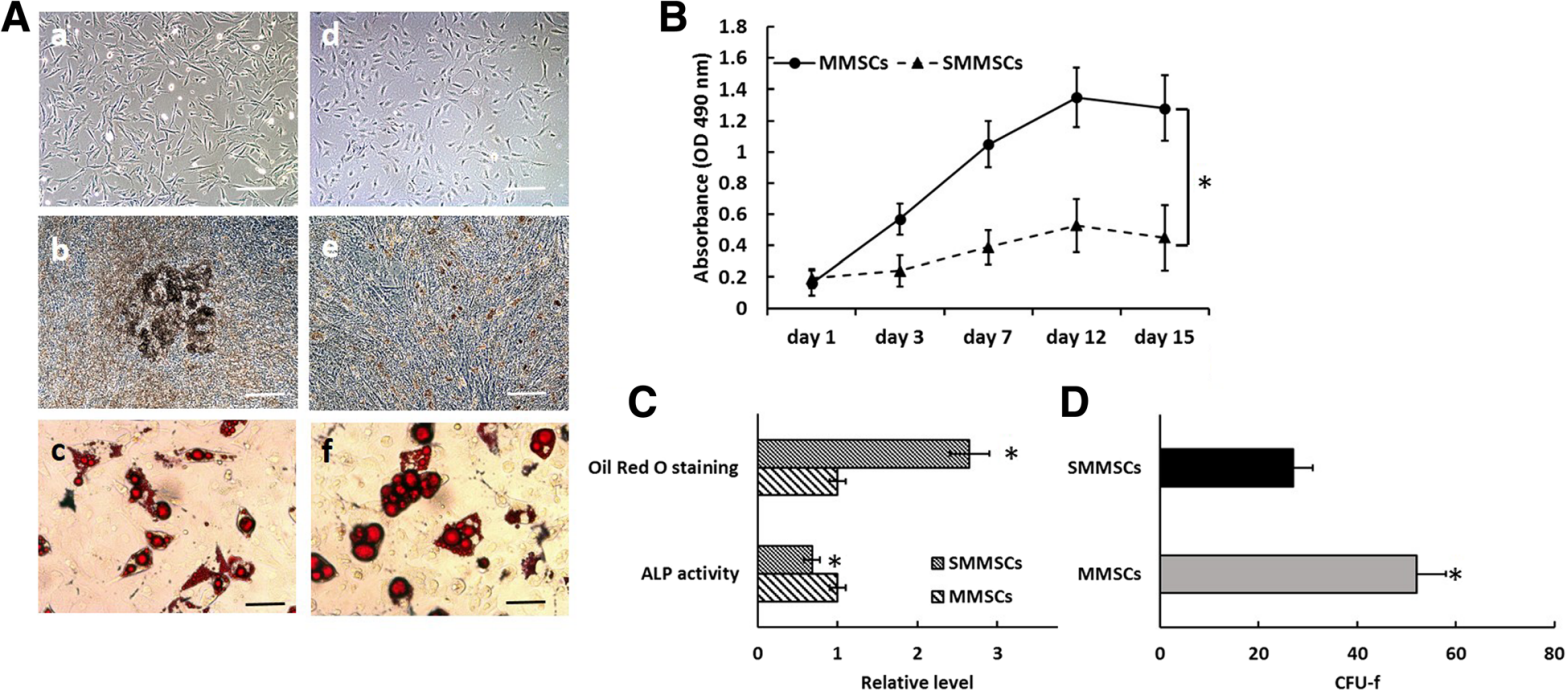
Proliferation and differentiation of MMSCs and SMMSCs in vitro. **A** The MMSCs cells (A[a]) and SMMSCs (A[d]) have similar typical fibroblast-like morphology after cultured 3 days in vitro. The differentiation was induced in MMSCs and SMMSCs for 2 weeks in vitro and progress of adipogenic and osteogenic differentiation was explored by cytochemistry. Osteogenic differentiation by ALP staining (b, e). Adipogenic differentiation was detected by Oil Red O staining (c, f). In contrast to MMSCs, efficient osteogenic differentiation could not be induced in SMMSCs [compare (b) vs. (e)]. The bars extend 100 μm (a-e) and 20 μm (c, f). **B** MTT assay was performed for cell proliferation after 1, 3, 7, 12 and 15 days culture with passages 5–8 cells of MMSCs and SMMSCs. The results show the cell growth rate of SMMSCs was significantly slower than MMSCs. **C** osteogenesis and adipogenic quantitative assay in MMSCs and SMMSCs groups. **D** CFU-f numbers in MMSCs and SMMSCs groups. After 15 days cultured in Mesenchymal Stem Cell Medium, colonies were stained with crystal violet and counted. Data are expressed as mean ± SD from all experiments, as indicated * *P* < 0.05

SMMSCs were isolated from SAM-P6 mouse. SMMSCs and MMSCs shared a similar fibroblast-like spindle shape, as could be seen in Fig. 1A [a, d]. Results of MTT analysis and colony formation assay indicated that cell growth rate of SMMSCs was significantly slower than that of MMSCs. Furthermore, such prominent difference persisted for 15 days of cell culture in Mesenchymal Stem Cell Medium (Fig. 1B and 1C, *p* < 0.05). It could be observed that, MMSCs and SMMSCs could undergo adipogenic differentiation and lipid accumulation in adipogenic induction medium, with adipogenic SMMSCs being more confluent than MMSCs. However, efficient osteogenic differentiation of SMMSCs could not be induced in osteogenic induction medium compared with MMSCs. Abilities of osteogenic and adipogenic differentiation of MMSCs and SMMSCs were evaluated and compared by ALP staining and Oil Red O staining in this research. It could be observed from Fig. 1A[b] and [e] that, ALP staining showed that MMSCs displayed a remarkably higher ALP level relative to SMMSCs after 14 days of subculture. In contrast, Oil Red O staining presented an enhanced ability of SMMSCs for lipid accumulation (Fig. 1A[c] and [f]). It can be found based on these results that under similar culture conditions, proliferation capacity of SMMSCs was retarded, which gave rise to their gradual loss of ability to differentiate into osteogenic lineage; however, their ability of adipogenic differentiation was still enhanced.

**Fig. 2:**
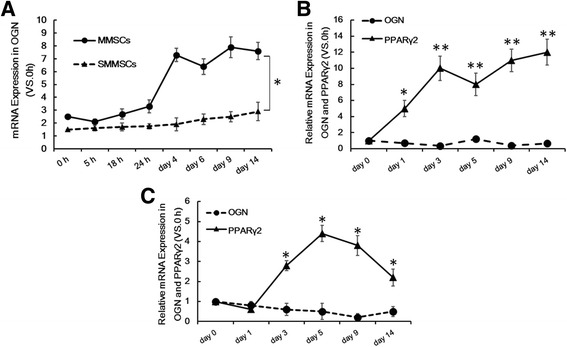
The mRNA expression of PPARγ2 and OGN in osteogenic or adipogenic differentiation of MMSCs and SMMSCs in vitro. **a** To investigate PPARγ2 and OGN in gene expression following differentiation, RT-qPCR was performed from 0 h to 14 days after induction of osteogenic differentiation in MMSCs and SMMSCs. **b** From 0 to 14 days after induction of adipogenic differentiation (PPARγ2 and OGN) in SMMSCs. **c** From 0 to 14 days after induction of adipogenic differentiation in MMSCs. Before differentiation (day 0) in SMMSCs served as controls as 1. Each assay was performed in three independent experiments. Data are expressed as mean ± SD from all experiments, as indicated * *P* < 0.05 and ** *P* < 0.01

OGN might play a marked role in bone formation by means of osteoblasts at the well-differentiated stage [14, 15]. mRNA expression levels of OGN from 0 to 14 days after the induction of osteogenic differentiation were detected by RT-qPCR, so as to investigate expression of OGN in SMMSCs with impaired osteogenesis. Compared with MMSCs, weak expression of OGN could be seen in SMMSCs after 3 days of osteogenic differentiation. (Fig. 2a [left], *p* < 0.05).

### mRNA expression level of PPARγ2 in SMMSCs was higher than that in MMSCs cultivated in adipogenic differentiation medium. Moreover, PPARγ2 might be negatively correlated with mRNA expression level of OGN

OGN could be stimulated by expression of Cbfa1 gene, which was a key gene of osteoblastogenesis [16]. PPARγ activity contributed to inhibiting osteoblastic maturation, as could be seen in changes in Runx2/Cbfa1 activity and OCN expression [17]. Expression levels of PPARγ2 and OGN in MMSCs and SMMSCs cultured in adipogenic differentiation medium were examined in this research. The results indicated that, compared with the control, expression of PPARγ2 was up-regulated for more than 12 times in SMMSCs from 0 to 14 days after the induction of adipogenic differentiation (day 0), as could be seen in Fig. 2b. However, only weak or absent expression of OGN could be seen in SMMSCs during the same time period. RT-qPCR assay in MMSCs after 0 to 14 days of induction of adipogenic differentiation also presented similar results, but expression of PPARγ2 was only up-regulated for 3–4 times compared with the control (day 0). mRNA expression level of OGN was quite low, which was down-regulated progressively. Conversely, mRNA expression level of PPARγ2, an adipogenic differentiation marker, had notably increased in adipogenic MMSCs (as was shown in Fig. 2b and c), indicating that the down-regulation of OGN might be involved in the functional increase in PPARγ2 expression. PPARγ2 might be negatively correlated with expression level of OGN in BMSCs during adipogenic differentiation.

### Effects of rosiglitazone on adipocyte or adipogenic differentiation in MMSCs and SMMSCs

**Fig. 3:**
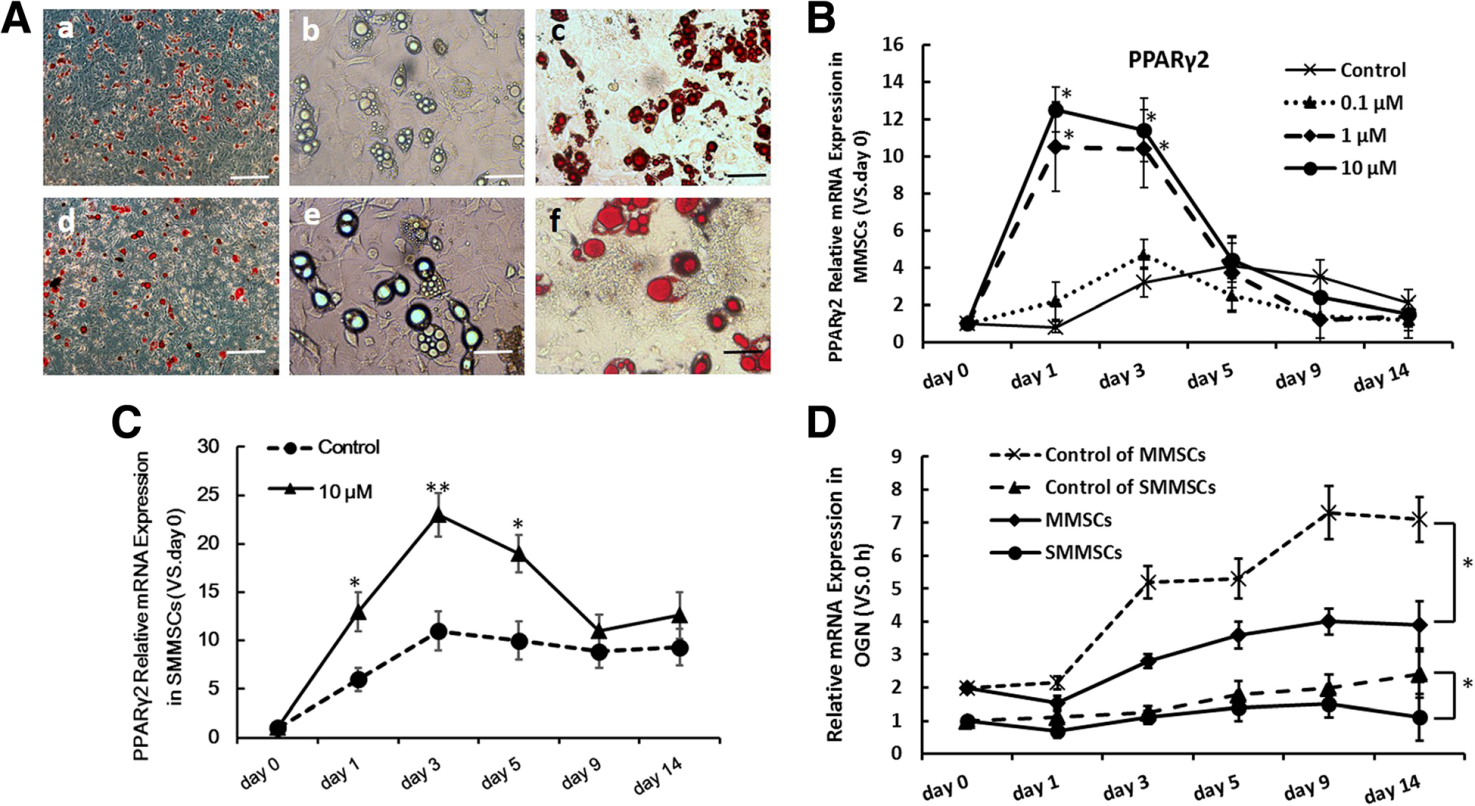
Influence of rosiglitazone on cell osteogenic or adipogenic differentiation in MMSCs and SMMSCs. **A** Adipogenic differentiation of MMSCs(a-c) and SMMSCs(d-f) treated with rosiglitazone (10 μM) for 14 days were detected by Oil red-O staining(a, c, d, f) or unstaining (b, e). The bars extend 100 μm (a, d) and 20 μm (b, c, e, f). **B** The expression of PPARγ2 in MMSCs were measured by RT-qPCR from 0 to 14 days in induction of adipogenic differentiation treat with different dose of rosiglitazone (0.1 μM, 1 μM and 10 μM). 0 μM rosiglitazone as control group., **C** The expression of PPARγ2 in SMMSCs from 0 to 14 days in induction of adipogenic differentiation treat with rosiglitazone (10 μM). **D** The expression of OGN in MMSCs and SMMSCs from 0 to 14 days in induction of osteogenic differentiation treat with rosiglitazone (10 μM). 0 μM rosiglitazone as control group. Before differentiation (day 0) served as controls as 1. Each assay was performed in three independent experiments. Data are expressed as mean ± SD from all experiments, as indicated * *P* < 0.05 and ** *P* < 0.01

MMSCs and SMMSCs were bipotential and were capable of differentiating into both osteoblast and adipocyte. Cells could mineralize the extracellular matrix in the presence of osteogenic differentiation stimuli. In contrast, the addition of a PPAR-γ ligand, such as rosiglitazone, to the adipogenic differentiation medium could induce fat accumulation and adipocyte differentiation, while suppressed the osteoblast phenotype of these cells, which was associated with down-regulation of OGN expression. As was presented in Fig. 3A, intracellular lipid droplet accumulation could be identified visually by oil red staining. The results indicated that compared with MMSCs group, which were treated by 10 μM rosiglitazone, SMMSCs could effectively promote fat droplet accumulation after 14 days of induced adipogenic differentiation culture.

Subsequently, effects of rosiglitazone on expression of phenotype specific gene markers in MMSCs were examined. It could be observed from Fig. 3B that, rosiglitazone affected expression of adipocyte-specific marker PPARγ2 at respective concentrations (0.1 μM, 1 μM and 10 μM, while 0 μM was treated as a control). Enhanced PPARγ2 expression could only be observed when concentration of rosiglitazone was greater than 1 μM. Compared with control group, expression of PPARγ2 mRNA could be up-regulated to 10–13 times in MMSCs (Fig. 3B) and 13–22 times in SMMSCs treated with 10 μM rosiglitazone (Fig. 3C).

Rosiglitazone could also effectively inhibit mRNA expression of OGN in MMSCs and SMMSCs. mRNA expression of OGN in MMSCs was reduced by 50% compared with control group after 5 days of induced osteogenic differentiation and rosiglitazone treatment. In addition, mRNA expression of OGN in SMMSCs was not even improved compared with day 0 (Fig. 3D). These results suggested that SMMSCs were more susceptible to the inhibition of rosiglitazone (as a PPAR-γ agonist), which made it more difficult to differentiate into osteoblasts in osteogenic induction culture. These data had validated that SMMSCs was a reliable model in vitro to investigate the roles of PPAR-γ2 and OGN in regulating differentiation of BMSCs into osteoblasts and adipocytes in senile mouse model.

### Forced OGN expression promoted osteogenic differentiation while inhibited adipocyte-specific marker expression in SMMSCs and MMSCs

**Fig. 4:**
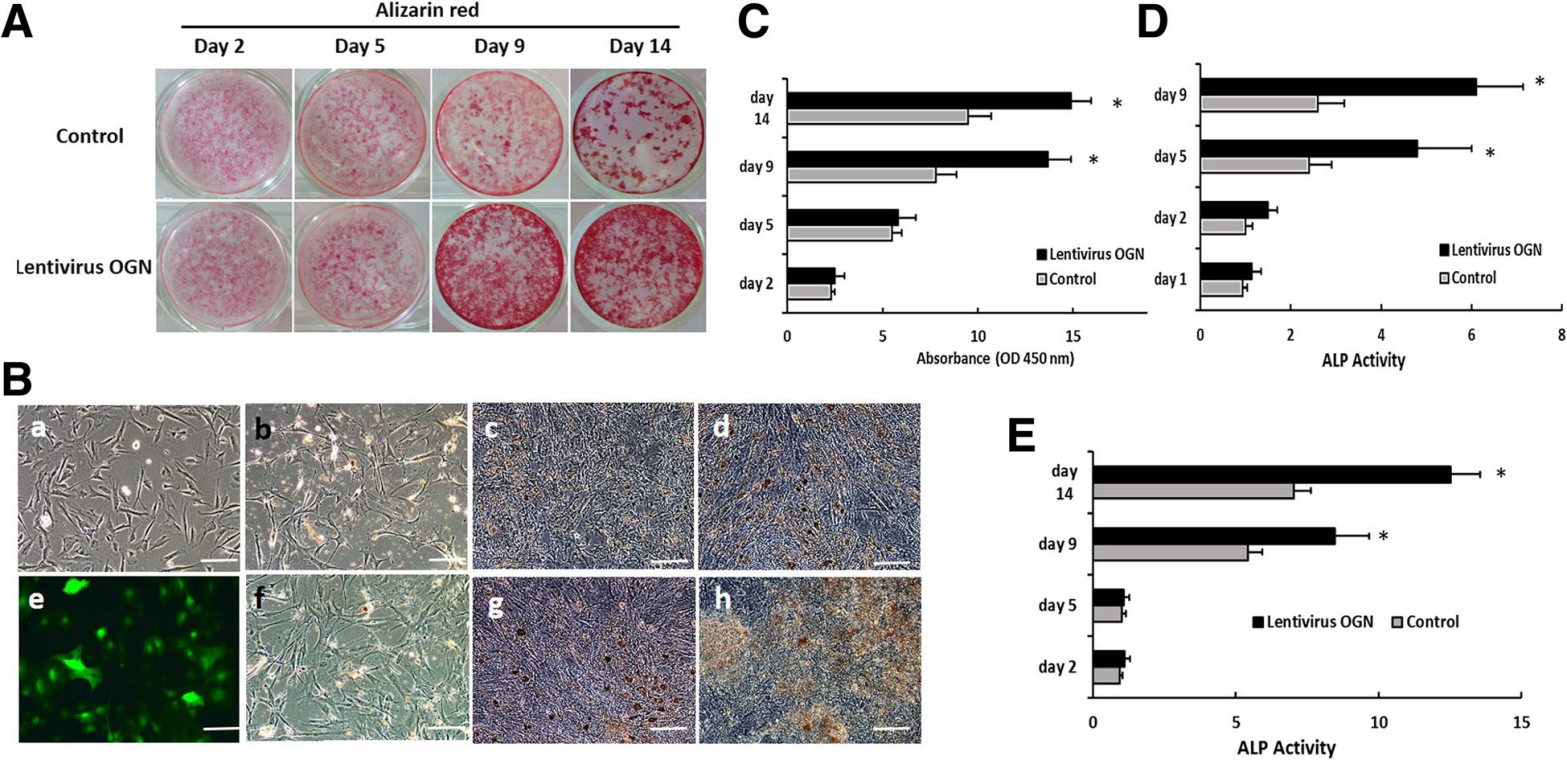
Influence of Lentivirus OGN on cell osteogenic differentiation in SMMSCs and MMSCs. **A** Alizarin-Red staining after osteogenic differentiation at 2, 5, 9 and 14 days in osteogenic medium. Compared with control group (A upper line), Lentivirus OGN enhanced the mineralization ability of osteoporotic SMMSCs (A lower line). **B** The differentiation was induced in SMMSCs (as control group, C [a-d]) and infected by Lentivirus OGN (as experimental group, C [e-h]) for 12 days in osteogenic medium and progress of osteogenic differentiation was explored by ALP staining at day 2 (b, f), 5 (c, g) and 9 (d, h). **C** Quantification of A was performed by optic density (O.D.) measurement at O.D. 450 nm (*n* = 3). **D** Quantification of B was performed by ALP activity. **E** Quantification of the differentiation was induced in MMSCs infected with Lentivirus OGN by ALP staining. Each assay was performed in three independent experiments. Data are expressed as mean ± SD from all experiments, as indicated * *P* < 0.05

Quantitative analysis of alizarin red staining in lentivirus OGN-infected SMMSCs was presented to be increased by an average of 75.6% (on day 5) and 56.8% (on day 14) in comparison with vector treatment cells (as control group) during osteogenic differentiation, as could be observed from Fig. 4A and C (*p* < 0.05).The same results were observed in MMSCs group (data not shown). In addition, the enhanced ALP staining demonstrated that infection of lentivirus OGN contributed to promoting mineralization of SMMSCs and MMSCs in the cultured osteogenic medium on day 2, 5, 9 and 14 (Fig. 4B). Quantification of ALP staining was performed by ALP activity, the results of which indicated that staining in lentivirus OGN-infected SMMSCs was increased by an average of 97.2% (on day 5) and 135% (on day 9) in comparison with the control(Fig. 4D). In MMSCs group, ALP staining was increased by an average of 56% (on day 9) and 77.8% (on day 14) in comparison with the control (Fig. 4E). All these findings revealed that OGN promoted the osteogenic differentiation of SMMSCs.

### Forced OGN expression down-regulated expression of adipocyte-specific marker while promoted that of osteoblast-specific gene marker by suppressing PPARγ2 expression

**Fig. 5:**
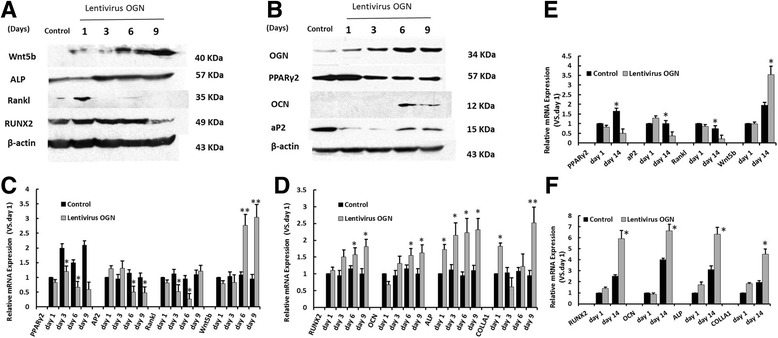
The mRNA and protein expression of osteogenic or adipogenic differentiation markers of SMMSCs and MMSCs infected by Lentivirus OGN compared with control groups. **a**, **b** Western blotting were employed to examine the protein expression of osteogenic markers genes RUNX2, Ocn, ALP, Wnt5b and adipogenesis marker genes AP2, PPARγ2 of SMMSCs infected by Lentivirus OGN compared with control groups at days 1, 3, 6 and 9. **c** The quantitative expression of adipogenesis markers gene AP2 and PPARγ2 were measured by RT-qPCR at days 1, 3, 6 and 9 after induction of adipogenic differentiation in SMMSCs infected by Lentivirus OGN. Uninfected cells as control group. **d** The quantitative expression of osteogenic markers gene RUNX2, Ocn, ALP, Colla1 and Wnt5b were measured by RT-qPCR at days 1, 3, 6 and 9 after induction of adipogenic differentiation in SMMSCs infected by Lentivirus OGN. Uninfected cells as control group. After differentiation 1 day served as controls as 1. **e** The quantitative expression of adipogenesis markers gene at days 1 and 14 after induction of adipogenic differentiation in MMSCs infected by Lentivirus OGN. **f** The quantitative expression of osteogenic markers gene at days 1 and 14 after induction of adipogenic differentiation in MMSCs infected by Lentivirus OGN. Each assay was performed in three independent experiments. Data are expressed as mean ± SD from all experiments, as indicated * *P* < 0.05 and ** *P* < 0.01

Adipocyte-specific markers as downstream PPARγ2 targets such as aP2, as well as Rankl, which was a ligand for osteoprotegerin that functioned as a key factor for osteoclast differentiation and activation, were examined by western blotting and RT-qPCR. In this way, the potential mechanism underlying the osteogenic differentiation mediated by OGN could be further explored. Compared with the vector group, PPARγ2 expression in lentivirus OGN-infected SMMSCs was down-regulated by 35.8% (on day 3, *p* < 0.05) and 51.3% (on day 6, p < 0.05) upon the stimulation of osteogenic differentiation, which were then increased to same levels as the control group at late phase of culture (Fig. 5c). As downstream PPARγ2 targets, expression of aP2 and Rankl in lentivirus OGN-infected SMMSCs presented similar results as PPARγ2, which were reduced mRNA (Fig. 5c) and protein (Fig. 5a and b) levels after 2 days of osteogenic differentiation cultured.

In contrast, compared with the vehicle group, mRNA levels of osteogenic marker genes (which were treated as downstream OGN targets), namely, runt-related transcription factor 2 (RUNX2), osteocalcin (OCN), wingless-type MMTV integration site family, member 5B (Wnt5b) were not changed at early phase. Nevertheless, RUNX2 was up-regulated at late phase by 36.5% (on day 6) and 81% (on day 9), OCN by 34.8% (on day 6) and 62% (on day 9) (Fig. 5d, *p* <0.05), and Wnt5b by 2.7-fold (on day 6) and 3-fold (on day 9) relative to vector group (Fig. 5c, p<0.01). ALP was the key enzyme utilized in the standard ALP staining method to detect mineralization of the matrix by osteoblasts. Production of controllable factor of type I collagen (Colla1) was increased rapidly at the beginning of induced osteogenic differentiation in lentivirus OGN-infected SMMSCs, as was shown in Fig. 5d. It was consistent with changes in protein expression levels of these osteogenic marker genes (Fig. 5a and b). The same results were observed in MMSCs group (Fig. 5e and f). All these results indicated that PPARγ2 was a positive promoter of adipogenesis as well as a negative regulator of osteoblastogenesis. OGN might play a distinct role in the retardative osteogenesis of osteoporotic SMMSCs through mediating the functional regulation of PPARγ2 expression.

## Discussion

Several conditions including senile osteoporosis are associated with bone loss, which is characterized by decreased osteoblastogenesis while increased adipogenesis in bone marrow [1, 18]. It supports the concept that various lineage-specific genes have exerted important roles during adipocyte, osteoblast and osteoclast differentiation, including RUNX2, OCN, ALP, Wnt5b, PPARγ2, aP2 and Rankl. It has been demonstrated in previous studies that PPARγ2 is up-regulated during aging, and that it is involved in adipocyte differentiation in vitro and in vivo as a key transcription factor [19].

Significant decreases in cell proliferation and osteogenic differentiation can be seen in senile osteoporotic SMMSCs; consequently, down-regulation of OGN may be associated with changes in osteoporotic SMMSCs. In addition, expression of PPARγ2 in SMMSCs and MMSCs within adipogenic differentiation medium is examined in this research, the results of which suggest that expression of PPARγ2 is outstandingly enhanced in SMMSCs and MMSCs in comparison with OGN. mRNA expression level of PPARγ2 in SMMSCs is higher than that in MMSCs cultivated in adipogenic differentiation medium. SMMSCs isolated from senile mouse gradually lose the ability to differentiate into the osteogenic lineage during osteoporosis, but adipogenic differentiation is still enhanced.

It is found in the present research that rosiglitazone, a PPARγ2 agonist, can activate expression of PPARγ2 and promotes PPARγ2 activity in adipocyte cultures. PPARγ2 can induce the differentiation of MMSCs and SMMSCs into adipocyte lineages; besides, it can negatively regulate osteoblast differentiation by means of suppressing expression of osteoblast specific transcription factor OGN. As is indicated in our findings, up-regulation of PPARγ2 is involved in inhibiting expression of osteogenic-related marker (such as OGN) in senile osteoporosis. SMMSCs are more susceptible to the inhibition of rosiglitazone, which adds to the difficulties to differentiate into osteoblasts in osteogenic induction culture.

Consequently, it is presumed that OGN may be of great significance to the retardative osteogenesis of senile osteoporotic SMMSCs through regulating expression of osteogenesis specific genes such as RUNX2, OCN, ALP and Wnt5b. Lentiviral vectors are applied to restore OGN expression in osteoporotic SMMSCs, so as to determine the effect of OGN on regulating osteogenic differentiation. It is found that the over-expression of OGN can up-regulate expression of osteogenesis-related markers (RUNX2, OCN, ALP and Wnt5b), while down-regulates that of genes characterizing phenotype of adipocyte (such as aP2 and PPARγ2), thus promoting osteogenic differentiation in osteoporotic SMMSCs. These results indicated that OGN-mediated signaling may plays an important role in regulating osteoblast differentiation and physiopathology of senile osteoporosis.

To gain further insight into the potential underlying mechanism of OGN, protein expression of these genes is investigated, including osteogenic marker genes RUNX2, OCN, ALP and Wnt5b as well as adipogenesis marker gene aP2 and osteoclast differentiation factor Rankl. Results of western blotting present that expression levels of osteogenic marker genes ALP, Wnt5b and OCN (the late stage osteogenic marker, which indicates bone formation [20]) are remarkably up-regulated in lentivirus OGN-infected SMMSCs at late stage of induced osteogenic differentiation (from day 3 to 9). Expression of another osteogenic marker gene RUNX2 is up-regulated rapidly at the beginning of osteogenic differentiation (from day 1 to 6), which is down-regulated on day 9. ALP is the key enzyme utilized in standard ALP staining method to detect matrix mineralization by osteoblasts, and ALP staining (Fig. 4c) supports the results of western blotting. As a member of the Wnt signaling pathway and Wnt receptor-ligand complex, Wnt5b has also been found to modulate different stages of osteogenic differentiation of hMSC when it is chemically induced in osteogenic differentiation [21]. In contrast, expression levels of adipogenesis marker genes aP2 and osteoclast differentiation factor Rankl are notably down-regulated in lentivirus OGN-infected SMMSCs compared with the control group, as is shown by results of western blotting. Besides, OGN is proved to inhibit expression of adipogenesis and osteoclast differentiation specific genes as well as lipid accumulation, thus preventing the transformation of BMSCs into adipocytes induced by the cultured differentiation. The same results were observed in MMSCs group. Taken together, these data suggest that OGN regulates the balance between adipogenesis and osteoblastogenesis in vitro in the manner of regulating Runx2. Furthermore, expression of OCN and ALP may be regulated through the Wnt5b/Wnt signaling pathway. Forced OGN expression by lentivirus-infected OGN contributes to increasing expression levels of RUNX2, OCN, ALP and Wnt5b expression, as well as bone formation, while decreasing expression of adipogenesis marker PPARγ2. It results in expression inhibition of adipocyte genes, such as adipogenesis-related genes aP2 and lipoprotein lipase (LPL) in the bone marrow, leading to increased bone mass. Therefore, PPARγ2 shows negative correlation with protein or mRNA expression levels of OGN. It can be found on the basis of these findings that PPARγ2 positively promotes adipogenesis while negatively regulates differentiation of BMSCs into osteoblast in vivo, implying that PPARγ2 is a negative regulator of bone mass. As a PPARγ2 antagonist, OGN helps to correct the imbalance between osteoblastogenesis and adipogenesis and displays a positive effect on bone mass, as compared with the up-regulated expression of osteogenic specific genes and osteoblast differentiation induced. In next steps, we will do further study on the appropriate manipulation of PPARγ2 expression by regulating OGN in senescence accelerated mouse prone/6 (SAM-P6), which is to the benefit of preventing bone loss in senile osteoporosis.

## Conclusion

In summary, PPARγ2 plays an important role in controlling the differentiation of marrow stromal cells into osteoblasts or adipocytes in senile osteoporosis, as is indicated in existing evidences. It is demonstrated in the present research that SAM-P6 mouse derived SMMSCs have a weakened capacity of osteogenic differentiation, which can be attributed to down-regulation of OGN. In addition, over-expression of OGN contributes to reversing the reduced capacity of osteogenic differentiation of SMMSCs. Furthermore, OGN can inhibit expression of adipogenesis marker gene aP2 and osteoclast differentiation factor Rankl by decreasing that of adipogenesis marker PPARγ2, which thereby promotes expression of osteogenic marker genes Wnt5b, RUNX2, OCN, ALP and Colla1, leading to osteoblast differentiation, as is suggested in a mechanistic analysis. Taken together, it is indicated by our findings that OGN may plays an important role in senile osteoporosis by regulate expression of osteogenic and adipogenesis genes, which may provide a potential target for therapeutic intervention for senile osteoporosis characterized by altered differentiation of BMSCs into osteoblasts and adipocytes.

## Abbreviations

BMSCs: Bone marrow-derived multipotent mesenchymal stromal cells
Colla1: Type I collagen
LPL: Lipoprotein lipase
MMSCs: Mouse mesenchymal stem cells
OCN: Osteocalcin
OGN: Osteoglycin
PPARγ: Peroxisome proliferators-activated receptor-γ
RUNX2: Runt-related transcription factor 2
SAM-P6: Senescence accelerated mouse prone/6
SMMSCs: Senescence accelerated mouse mesenchymal stem cells
Wnt5b: Wingless-type MMTV integration site family, member 5B
